# RETRACTION: The Effect of Different Wound Dressing Materials Used in Postoperative Treatment of Wounds After Total Hip Arthroplasty and Total Knee Arthroplasty: A Meta‐Analysis

**DOI:** 10.1111/iwj.70425

**Published:** 2025-03-26

## Abstract

**RETRACTION:** YuanY. , LiJ. , WangK. , ZhengG. , and ChaiS. , “The Effect of Different Wound Dressing Materials Used in Postoperative Treatment of Wounds After Total Hip Arthroplasty and Total Knee Arthroplasty: A Meta‐Analysis,” International Wound Journal 19, no. 8 (2022): 2107–2114, 10.1111/iwj.13816.35470964 PMC9705168

The above article, published online on 26 April 2022, in Wiley Online Library (http://onlinelibrary.wiley.com/), has been retracted by agreement between the journal Editor in Chief, Professor Keith Harding; and John Wiley & Sons Ltd. Following an investigation by the publisher, all parties have concluded that this article was accepted solely on the basis of a compromised peer review process. The editors have therefore decided to retract the article. The authors did not respond to our notice regarding the retraction.

**RETRACTION:** Y. Yuan , J. Li , K. Wang , G. Zheng , and S. Chai , “The Effect of Different Wound Dressing Materials Used in Postoperative Treatment of Wounds After Total Hip Arthroplasty and Total Knee Arthroplasty: A Meta‐Analysis,” International Wound Journal 19, no. 8 (2022): 2107–2114, 10.1111/iwj.13816.35470964 PMC9705168

The above article, published online on 26 April 2022, in Wiley Online Library (http://onlinelibrary.wiley.com/), has been retracted by agreement between the journal Editor in Chief, Professor Keith Harding; and John Wiley & Sons Ltd. Following an investigation by the publisher, all parties have concluded that this article was accepted solely on the basis of a compromised peer review process. The editors have therefore decided to retract the article. The authors did not respond to our notice regarding the retraction.

